# Rapid screening for neglect following stroke: A systematic search and European Academy of Neurology recommendations

**DOI:** 10.1111/ene.15381

**Published:** 2022-06-30

**Authors:** Margaret Moore, Elise Milosevich, Roland Beisteiner, Audrey Bowen, Matthew Checketts, Nele Demeyere, Helena Fordell, Olivier Godefroy, Jan Laczó, Timothy Rich, Lindy Williams, Kate Woodward‐Nutt, Masud Husain

**Affiliations:** ^1^ 6396 Department Experimental Psychology University of Oxford Oxford UK; ^2^ Queensland Brain Institute University of Brisbane Brisbane Qld Australia; ^3^ Department of Neurology Medical University of Vienna Vienna Austria; ^4^ Geoffrey Jefferson Brain Research Centre Manchester Academic Health Science Centre Northern Care Alliance and University of Manchester Manchester UK; ^5^ Division of Psychology and Mental Health Faculty of Biology, Medicine, and Health University of Manchester Manchester Academic Health Science Centre Manchester UK; ^6^ Neurosciences Umeå University Umeå Sweden; ^7^ Department of Neurology and Laboratory of Functional Neurosciences and Pathologies Amiens University Medical Center Jules Verne University of Picardy Amiens France; ^8^ Memory Clinic Department of Neurology Second Faculty of Medicine and Motol University Hospital Charles University Prague Czechia; ^9^ International Clinical Research Center St Anne's University Hospital Brno Brno Czechia; ^10^ 158368 Kessler Foundation West Orange New Jersey USA; ^11^ Physical Medicine and Rehabilitation Rutgers University Newark New Jersey USA; ^12^ 1067 Cognitive Aging and Impairment Neurosciences Lab University of South Australia Adelaide SA Australia; ^13^ Research and Innovation Northern Care Alliance National Health Service Group Salford UK; ^14^ 6396 Nuffield Department of Clinical Neurosciences University of Oxford Oxford UK

**Keywords:** cognitive impairments, diagnostic screening programs, hemispatial neglect, stroke

## Abstract

**Background and purpose:**

Unilateral neglect is a common cognitive disorder following stroke. Neglect has a significant impact on functional outcomes, so it is important to detect. However, there is no consensus on which are the best screening tests to administer to detect neglect in time‐limited clinical environments.

**Methods:**

Members of the European Academy of Neurology Scientific Panel on Higher Cortical Functions, neuropsychologists, occupational therapists, and researchers produced recommendations for primary and secondary tests for bedside neglect testing based on a rigorous literature review, data extraction, online consensus meeting, and subsequent iterations.

**Results:**

A total of 512 articles were screened, and 42 were included. These reported data from 3367 stroke survivors assessed using 62 neglect screens. Tests were grouped into cancellation, line bisection, copying, reading/writing, and behavioral. Cancellation tasks were most frequently used (97.6% of studies), followed by bisection, copying, behavioral, and reading/writing assessments. The panel recommended a cancellation test as the primary screening test if there is time to administer only one test. One of several cancellation tests might be used, depending on availability. If time permits, one or more of line bisection, figure copying, and baking tray task were recommended as secondary tests. Finally, if a functional and ecological test is feasible, the Catherine Bergego Scale was recommended. Overall, the literature suggests that no single test on its own is sufficient to exclude a diagnosis of neglect. Therefore, the panel recommended that multiple neglect tests should be used whenever possible.

**Conclusions:**

This study provides consensus recommendations for rapid bedside detection of neglect in real‐world, clinical environments.

## Graphical abstract

An expert panel of neurologists, neuropsychologists, occupational therapists, and researchers provides consensus recommendations for neglect screening in real‐world clinical environments. Overall, cancellation tasks are recommended as first‐line screens, with line bisection, figure copying, and baking tray tasks and Catherine Bergego Scale recommended as secondary screens. The panel recommends using multiple screening tools whenever time allows.

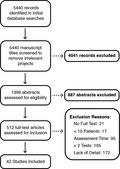

## INTRODUCTION

Unilateral neglect is a common poststroke cognitive impairment characterized by consistently lateralized spatial attentional deficits [1, 2]. The occurrence of neglect acts as a key predictor of poor long‐term recovery following stroke, with neglect patients experiencing lower quality of life and demonstrating reduced motor/functional abilities as well as higher levels of mood disorders than patients without neglect [3, 4, 5, 6]. It is therefore critically important to detect neglect impairment to provide important prognostic indicators and to facilitate targeted rehabilitation approaches.

Currently, a wide range of methods are employed to screen for neglect within clinical environments [7]. Checketts et al. [8] conducted a large‐scale, international survey aiming to identify common screening methods in clinical practice. Cognitive tasks were found to be the most popular form of neglect assessment (used by 82% of those responding to the survey), followed by functional assessments (used by 80%) [8]. A similar, Danish nationwide study conducted by Evald et al. [9] reported that subjective clinical observations were the most common assessment method, used by 90% of those surveyed, whereas pen‐and‐paper cognitive tasks were used by 49%. However, a wide range of individual tests were included within each of these reported assessment type categories. For example, Checketts et al. [8] reported on 14 different neuropsychological tests, including line bisection, copying, and cancellation tasks, within the “cognitive assessments” category. Similarly, 11 different screening methods were included within the “functional assessments” category, ranging from unstructured observations to standardized functional assessment tools [8]. Given this variation, it is important for clinicians to have access to recommendations for methods to detect neglect.

Previous investigations have come to varying conclusions on whether it is better to use observational or pen‐and‐paper neglect screening methods [10, 11, 12], whether specific pen‐and‐paper tasks represent valid methods for detecting impairment [13, 14], and what is the single best method for detecting neglect in clinical environments [15, 16, 17]. Overall, the existing literature has strongly suggested that, ideally, neglect should be screened for by comparing performance across a battery of independent and multimodal neglect assessments [15, 18, 19, 20, 21]. However, given the time and resource constraints associated with real‐world clinical environments, this practice is generally not feasible. It is therefore crucial to determine which neglect screening methods should be used in cases where real‐world time and resource constraints allow for only one or a few screening tests.

An important issue for any recommendations regarding best tests to use for screening is that there is no objective gold standard against which tests can be compared. Furthermore, because there can be dissociations in the nature of neglect (e.g., egocentric vs. allocentric [22, 23] or personal vs. extrapersonal [14, 24, 25]), some tests might, in theory, be able to detect only certain forms of neglect. It is important to detect different neglect subtypes, as previous research has demonstrated that these subtypes are dissociable and differentially associated with long‐term recovery outcomes [5, 15, 16]. In addition, some patients show neglect during everyday functional tasks but perform normally on pen‐and‐paper tests of neglect, particularly due to testing/practice effects that can accompany repeated assessment. Thus, there are many factors in addition to reported number of neglect cases that must be considered to identify the best assessment methods. Moreover, neglect screening methods must be practical, inclusive, time‐efficient, and easy to administer without specialist equipment or training. Given the diverse factors that must be considered when assessing the practicality of any single neglect test, there is a clear need for the existing literature to be systematically analyzed to identify the individual assessment methods that are most strongly supported by evidence.

The present study aims to review the existing literature and produce expert consensus recommendations for the individual tests that should be used to screen for neglect impairment within real‐world clinical environments. First, a systematic literature search was performed to identify previous studies that compared neglect assessment methods. This literature was then reviewed by an interdisciplinary expert panel consisting of professional neurologists, neuropsychologists, occupational therapists, and researchers to identify the best neglect screening methods. These recommendations were then categorized into primary recommendations for conditions in which time allows for only a single neglect screening test and secondary recommendations where additional tests are possible. Overall, this project provides expert recommendations aiming to optimize current clinical neglect screening practice.

## METHODS

### Systematic literature search

A systematic literature search was conducted to identify previous studies that compared neglect screening tests. The search protocol employed in this study has been made openly available on the Open Science Framework (https://osf.io/fzmde/). PubMed, Embase, PsychINFO, Scopus, Web of Science, and the Cochrane Library were searched from inception to 30 April 2020 using MeSH (medical subject heading) terms related to stroke, neglect, and neuropsychological assessment. Articles were considered for inclusion if they reported observational studies or randomized controlled trials including human participants older than 18 years assessed within 3 months of a clinically confirmed diagnosis of stroke. Projects were excluded from consideration if they were not written in English, reported data from fewer than 10 adult patients with stroke, or were not available in full text. Finally, studies were excluded if they did not report the results of at least two systematic and independent neglect screens in sufficient detail to facilitate comparisons.

Articles surviving this process were then reviewed to extract publication details, sample characteristics, neglect tests employed, and comparative frequency of neglect impairment according to each test. Because we were interested in screening (avoiding false negatives), when articles reported overall frequency of impairment on several neglect measures, we selected the single test yielding the highest number of possibly impaired patients.

The resulting data were then reviewed by a team of clinical and research neurologists, neuropsychologists, and occupational therapists to identify the best tests for screening for neglect in clinical environments. The relevance of each considered test was evaluated on the basis of reported number of neglect cases detected, ease of use for examiners and participants, and time efficiency. The panel also considered whether each tool was openly available, as the costs associated with restricted access tests might be prohibitive for many users. To reach a formal consensus, each panel member reviewed the shortlisted papers to evaluate quality of evidence. The panel then held a meeting in which each identified test was sequentially discussed and members voted on whether they recommended each test. In cases where the vote was split, the panel continued discussion until agreement was reached. The results of this discussion were transcribed and evaluated by each panel member for approval prior to finalization.

The recommended tests were divided into primary and secondary categories. Primary tests represent assessments that were unanimously agreed to represent the best options for a time‐efficient neglect screening assessment within a clinical environment. Secondary tests include assessments that can be administered in addition to the recommended primary tests to provide additional details pertaining to the type, severity, and potential impact of neglect impairment.

## RESULTS

### Systematic search results

**FIGURE 1:**
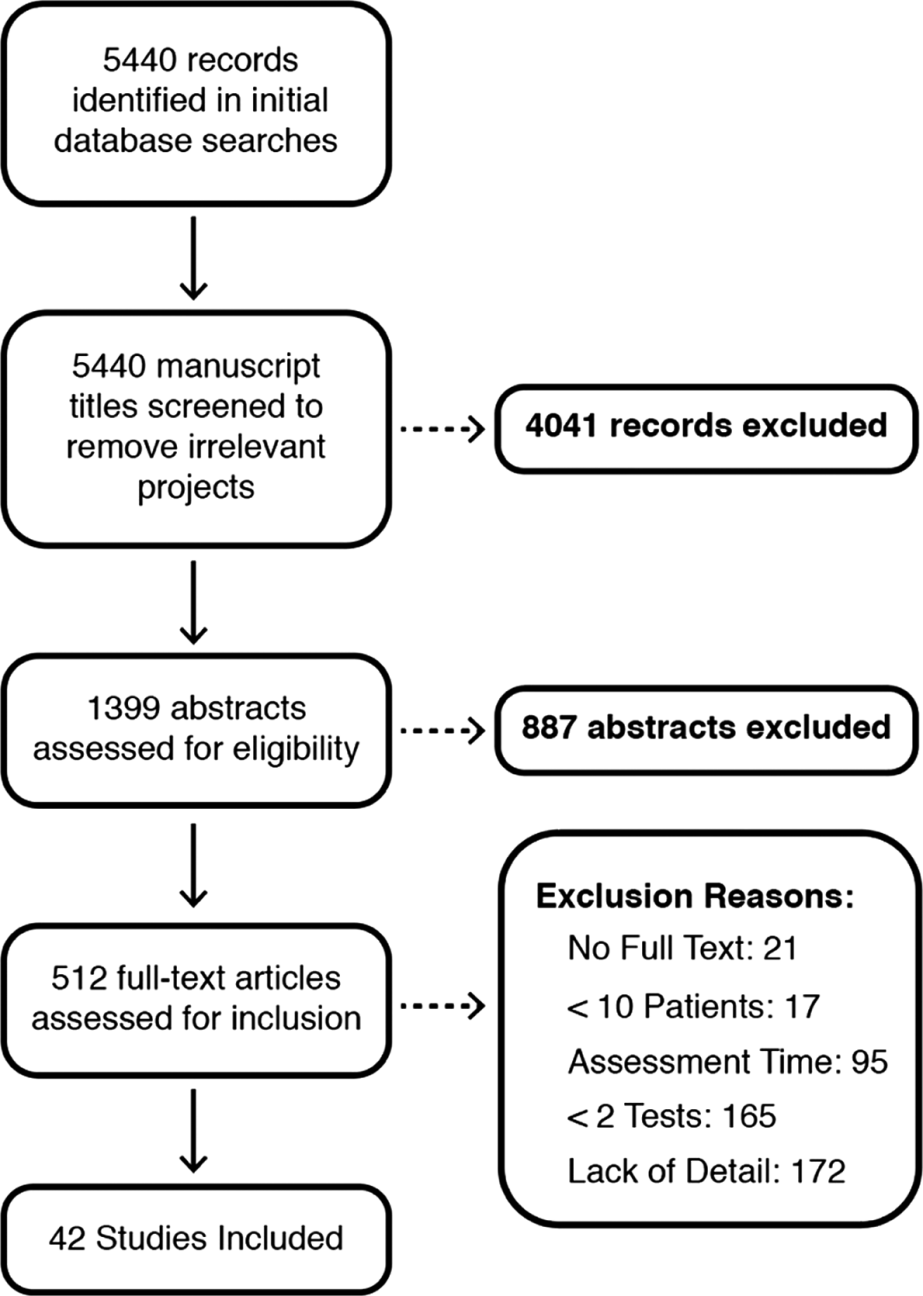
Visualization of systematic literature search and exclusions at each stage

Systematic literature review yielded a total of 42 articles meeting all inclusion criteria (Table 1). The process as well as the number of articles excluded at each stage is presented in Figure 1. Of the included projects, 21 studies included only right hemisphere patients, one included only left hemisphere patients, 19 recruited patients regardless of lesion location, and three provided insufficient information to determine lesion side. Overall, 17 studies were conducted in rehabilitation units, 13 were conducted on acute stroke wards, 3 were multicenter studies, 2 recruited from outpatient locations, and 7 did not report study setting. Finally, 18 studies recruited patients within the acute phase (<30 days poststroke), 9 recruited subacute patients (31–90 days), 1 included only chronic patients (>90 days), 10 included patients recruited at a mix of these time points, and 7 studies did not report recruitment time. In total, 28 studies reported recruiting consecutive samples.

**TABLE 1:** Summary of analyses conducted in studies identified within the literature review

Paper	*n*	Cancellation	Bisection	Copying	Reading/writing	Behavioral	Test detecting highest *n*
Alqahtani [26]	165	1*	1	1			Bells test [27]
Apperlos et al. [28]	282	2	1			3*	Baking tray task [29]
Azouvi [30]	50	2		2	1*	1	Reading test [31]
Azouvi et al. [ 32]	206	1	1	2	2	3*	CBS [10]
Azouvi et al. [10]	83	1		1		1*	CBS [10]
Azouvi et al. [18]	284	1*	1	2	2		Bells test [27]
Bachman et al. [ 33]	50	2*	1			1	Letter cancellation [34]
Bailey et al. [35]	107	1*	2	2		2	BIT star cancellation [36]
Bailey et al. [15]	168	1*	1			1	BIT star cancellation [36]
Beis et al. [37]	89	1*	1	2			Bells test [27]
Berti et al. [38]	34	2*		2			Bells test [27]
Binder et al. [39]	34	1	2*				BIT bisection [36]
Brunila et al. [40]	34	3*	1	1			BIT star cancellation [36]
Chiba et al. [41]	14	1*		1			Albert's test [42]
Cumming et al. [43]	71	1				1*	NIHSS [44]
Cunningham et al. [45]	50					2*	CBS [10]
Demeyere et al. [46]	208	2*		1			OCS cancellation test [46]
Edmans & Lincoln [ 17]	150	1		2*			Word copying [47]
Fordell et al. [48]	31	2	2	1		2*	Baking tray task [29]
Friedman [49]	41	1*	1	2			BIT star Cancellation [36]
Grattan & Woodbury [50]	12	5	1		5	3*	NAT [51]/VRLAT [52]
Halligan et al. [53]	80	3*	1	1		4	BIT star cancellation [36]
Kaufmann et al. [54]	15	3*	1				Bells test [27]
Kettunen et al. [55]	37	3*	1	1			BIT star cancellation [36]
Kinsella et al. [56]	40	2*	1			2	Shape cancellation [57]
Klinke et al. [58]	23	2*	1	1		1	BIT star cancellation [36]
Lindell et al. [59]	34	5*	2	1		2	Shape cancellation [57]
Lopes et al. [60]	102	3*	1	1			BIT star cancellation [36]
Lundervold et al. [61]	13	3	2	1*			BIT copying [28]
Marsh & Kersel [62]	27	2*	1				BIT star cancellation [36]
Moore et al. [11]	428	1*				1	OCS cancellation [29]
Park et al. [63]	45	2	2*	1			Letter line bisection [64]
Rousseaux et al. [65]	15	1	1*			2	BIT bisection [28]
Sperber & Karnath [14]	180	1*		1			Bells test [28]
Stone et al. [66]	44	3*		1	2		BIT star cancellation [36]
Tatuene et al. [67]	98	1*	1			1	Gap detection test [68, 69
Upshaw et al. [70]	20	1				1*	Eye‐tracking (original)
Van der Stigchel & Nijboer [71]	73	1	1*				Line bisection (unspecified)
Vanier et al. [72]	47	2*					Bells Test [28]
Veronelli et al. [73]	22	3	1*	2	1		Line bisection (original)
Welmer et al. [74]	115	1*					Letter cancellation [30]
Yin et al. [75]	30	1	1*	1		]	Line bisection [35]
Overall	3367	25/41	6/28	2/25	1/6	8/19	BIT Star cancellation [36]

Note

For each study, the number of tests used within each category is noted. The tests that were found to detect the highest frequency of neglect cases are reported and the test category containing each of these tests is marked with an asterisk (*); *n* denotes number of patients with stroke included. Citations in the first column refer to included papers while citations in the last column are references for specific tests used by the included papers.

Abbreviations: BIT, Behavioral Inattention Test; CBS, Catherine Bergego Scale; NAT, Naturalistic Attention Test; NIHSS, National Institutes of Health Stroke Scale; OCS, Oxford Cognitive Screen; VRLAT, Virtual Reality Lateralised Attention Test.

Cumulatively, these studies report data from 3367 stroke survivors assessed using 62 different neglect screening tools. These screening tools can be grouped into cancellation, line bisection, copying, reading/writing, and behavioral test categories. Cancellation tasks were found to be the single most frequently used assessment class (used in 97.6% of included studies), followed by bisection (used in 66.7%), copying tasks (used in 60%), behavioral tasks (used in 45.3%), and reading/writing assessments (used in 14.3%). Overall, cancellation tasks most frequently resulted in the highest positive screening rates (most cases reported within 59.5% of studies), followed by behavioral (reported in 19.0%), bisection (reported in 14.2%), copying (reported in 4.8%), and reading/writing tests (reported in 2.4%; Table 1).

Within the 20 studies that conducted comparisons across several different cancellation tasks, the Star Cancellation from the Behavioral Inattention Test (BIT) [36] was most frequently found to be the best cancellation task (12/20), followed by the Bells Test [27] (4/20). However, it is important to note that older cancellation tests (e.g., BIT Star Cancellation [36]) have been included in more previous analyses than newer cancellation tests (e.g., Oxford Cognitive Screen [OCS] [29]), so these findings may partially be explained by the test's popularity and history rather than its underlying sensitivity. For this reason, further direct, head‐to‐head studies are needed to evaluate assessment quality.

### Primary consensus recommendations

The results of the included studies were first evaluated by a panel of expert neurologists, neuropsychologists, and researchers to identify the screening tests recommended for use in clinical situations. Overall, the existing literature strongly suggests that no single neglect screening test on its own is sufficient to exclude a diagnosis of neglect. The panel therefore recommends that whenever possible, multiple neglect screening tests should be employed. However, recommendations were also made for real‐world conditions in which time constraints often allow only one or a few tests to be performed.

In line with the included literature, a consensus recommendation was made that a cancellation task should be used to perform primary neglect assessment. Overall, cancellation tasks that have been experimentally validated in rigorous, large‐scale investigations (e.g., BIT Cancellation, Bells Test, OCS Cancellation, Birmingham Cognitive Screen [BCoS] Apples Cancellation) are preferred. This use of normative data is crucial, as even healthy controls may exhibit some small degree of spatial attentional biases [76, 77]. However, the panel noted that many popular and validated cancellation tasks (e.g., BIT Cancellation [36], Rivermead Perceptual Assessment Battery [47]) are not openly available. Furthermore, cancellation tasks with a comparatively low stimulus density (e.g., Albert's Test, Coin Selection) may have a lower probability of detecting neglect than those with higher density [16, 78], whereas tasks with a very high complexity (e.g., Mesulam Shape Cancellation) may prove to be too difficult for many patients with acute stroke to complete. Similarly, tasks that employ language‐based cancellation stimuli (e.g., Letter Cancellation Tests) may be confounded by unrelated, comorbid letter or word identification deficits.

Finally, neglect is not a unitary syndrome; different patients exhibit egocentric or allocentric attentional deficits [5, 23, 79, 80]. For this reason, cancellation tests that can distinguish between egocentric and allocentric neglect [29] are useful, if these are accessible.

Reading‐ and writing‐based neglect assessments were not recommended as primary neglect assessments, because assessment of function with these tasks might be precluded by comorbid language and fine‐motor deficits in a substantial portion of the stroke population [81, 82, 83, 84]. Neglect assessments based on behavioral observations were not recommended for primary use due to the documented susceptibility to expectation biases due to lesion location [11]. Additionally, behavioral observation is not ideal for rapid, first‐line assessment due to the potentially time‐consuming need to observe patients interacting with real‐world environments (as in the Catherine Bergego Scale, discussed below) [10].

Although line bisection tasks are commonly employed to quantify neglect, the results of some studies suggest that these tasks do not represent a valid method for detecting neglect impairment [14]. Bisection tests may measure a different behavioral construct than cancellation and copying tests [14] and yield fine‐grained continuous behavioral metrics that are vulnerable to confounding bias from comorbid fine‐motor impairments, hemianopia, and optic ataxia [19]. For these reasons, line bisection tasks were not recommended for neglect screening if only one test can be used. Copying tests were also not considered to be suitable as the primary test for neglect screening due to potential interference from comorbid motor and cognitive deficits [85, 86] as well as comparative difficulty in calculating quantitative neglect impairment scores [86]. Finally, the National Institutes of Health Stroke Scale (NIHSS) is commonly used as a first‐line neglect screen in clinical environments [1]. However, previous literature has demonstrated that this screen is <30% sensitive compared to cancellation tasks, is highly susceptible to clinician expectation biases, and commonly misdiagnoses visual field impairment as neglect [1, 87]. For these reasons, the NIHSS was not recommended to be used for neglect assessment.

Overall, the expert panel unanimously agreed that a form of cancellation task should be the first choice of neglect assessment if there is time for only one test (Table 2). If available, established measures such as the BIT Sar Cancellation [36] and Bells Test [27] can be used. The OCS Hearts Cancellation Test [29] and the BCoS Apples Cancellation Task [16, 69] are also recommended as primary neglect assessment methods within clinical environments. The latter tests are openly available, and also provide a potential means to distinguish between egocentric and allocentric neglect.

**TABLE 2:** Summary of recommendations for primary and secondary neglect screening

Consensus recommendations for neglect screening		
	Time, min	Test access
Primary recommendation		
One of the following cancellation tests		
BIT Star Cancellation Task (Wilson et al., 1987 [36])	<5	www.pearsonclinical.co.uk
Bells Cancellation Test (Gauthier et al., 1989 [27])	<5	https://strokengine.ca ^a^
OCS Hearts Cancellation Test (Demeyere et al., 2015 [29])	3	https://www.ocs‐test.org ^a^
BCoS Apples Cancellation Task (Bickerton et al, 2011 [16])	<5	https://www.cognitionmatters.org.uk/bcos.php
Secondary recommendations		
If time permits & test available consider one/more of		
Figure copying (e.g., Wilson et al., 1987 [36])	<5	www.pearsonclinical.co.uk
Line bisection (e.g., Wilson et al., 1987 [29])	<5	www.pearsonclinical.co.uk or https://strokengine.ca ^a^
Baking tray task (e.g., Tham, 1996 [29])	<5	https://health.utah.edu/sites/g/files/zrelqx131/files/files/migration/image/bakingtray.pdf ^a^
Functional/ecological assessment of neglect		
If longer assessment of everyday activity possible		
Catherine Bergego Scale (Azouvi et al., 2003 [10])	30	https://www.tandfonline.com/doi/pdf/10.1080/713755501?needAccess=true ^a^

Note

Importantly, these recommendations are for a rapid initial screening. Formal neglect diagnosis should be based on the results of multiple, distinct neglect tests.

Abbreviations: BCoS, Birmingham Cognitive Screen; BIT, Behavioral Inattention Test; OCS, Oxford Cognitive Screen.

^a^ Screening tools that are freely available.

### Secondary consensus recommendations

Due to its heterogeneity, previous research has shown that neglect should be screened for by comparing performance across several, independent neglect assessments [18, 19, 21, 26]. Where time allows for more than one test, clinicians should conduct additional neglect assessments. Therefore, the included literature was analyzed by the expert panel to provide secondary recommendations for additional neglect assessments.

Three types of test were recommended by the panel as adjuncts to a cancellation test. Despite discussed limitations associated with using manual line bisection tasks to assess visuospatial neglect impairment, some previous studies have identified patients demonstrating neglect on bisection, but not cancellation tasks [64, 65, 72]. Prior research has suggested that manual line bisection tasks may be most appropriate for detecting co‐occurrence between visual field deficits and egocentric neglect [88]. In cases where bisection tasks are used, clinicians should aim to employ standardized manual bisection tasks with published normative performance thresholds (e.g., Wilson et al. [36]) rather than improvised, original tasks. This use of normative data is critically important, as controls have also been found to exhibit small biases in line bisection tests [76, 77]. Given some of the limitations associated with using bisection tests to quantify neglect [14], the panel recommended that they be used as secondary assessments, but biased performance on bisection tests alone should not be considered sufficient evidence to detect neglect impairment.

Next, figure copy tasks [89] were also recommended for secondary neglect assessment. These are easy to administer and improvise within clinical environments. Copying and drawing tests may help provide insight into some components of neglect not clearly assessed by standard cancellation tests (e.g., drawing from memory for representational neglect) [90]. However, past research has demonstrated that these tasks might detect a lower frequency of neglect than cancellation tasks and are reliant on subjective interpretations of impairment rather than quantitative scoring systems [90]. As in cancellation tests, a wide range of copying‐based neglect assessments are in use. In general, copying tasks that display multiple stimuli on the horizonal axis and are able to distinguish between egocentric and allocentric neglect (e.g., scene copying tasks) are more informative than those that employ simpler stimuli (e.g., daisy copying) [90, 91].

Finally, baking tray tasks [92] were recommended as a secondary neglect assessment method. In this task, patients are asked to arrange items evenly across a tray as if they were “buns on a baking tray” [92]. Patients with egocentric neglect have been found to demonstrate a clear spatial bias on this task, crowding all items onto one side of the tray area [15, 92]. Baking tray tasks are easy to improvise within clinical environments by making a “tray” and “items” with standard, normed dimensions (e.g. Facchin et al. [93]). This task has been demonstrated to be highly sensitive to neglect if it is possible to perform it in a clinical environment [15,28, 29].

### Functional/ecological evaluation of neglect recommendation

The panel also acknowledged that if time is available and if the patient's condition allows, functional/ecological tests should be performed. If feasible, the Catherine Bergego Scale [10] was recommended. This is a functional observation checklist that provides a naturalistic assessment of how neglect impairment manifests in real‐world activities, such as grooming and navigation. A standardized protocol for administering this assessment has been developed [94]. It can outperform many pen‐and‐paper assessments in detection of neglect [94. However, it is generally not feasible to observe all the behavior necessary to accurately complete the checklist within very brief initial clinical assessments, and the assessment requires experienced observers. Nevertheless, it can be a useful adjunct to rapid bedside assessments.

The Dublin Extrapersonal Neglect Assessment [45] also provides a highly naturalistic and informative assessment of how neglect impairment impacts on real‐world function. In this test, patients are asked to navigate through a hallway and locate a series of signs placed by the examiner [45]. However, this requires patients to mobilize (or be assisted) down a hallway, which is often not possible, particularly in hyperacute stroke. For this reason, this might be better suited for use in a slightly later stage of the stroke pathway (e.g., occupational therapy assessment for discharge planning). Furthermore, because this test has not been extensively deployed, the panel did not recommend its routine use.

## DISCUSSION

It is critically important to screen for neglect in patients with stroke, as the occurrence of this cognitive deficit has been found to be a key predictor of recovery outcomes [3, 4, 5, 6, 16]. This study aimed to evaluate existing literature comparing different neglect screening methods to provide consensus recommendations for how to detect neglect in real‐world clinical environments. The analysis considered a test's reported utility in detecting neglect (number of patients screening positive in a sample), practicality, inclusiveness, and availability. Importantly, no single neglect screening test should be considered sufficient to support a formal neglect diagnosis. However, the panel provided recommendations for real‐world clinical situations in which a full neglect test battery may not be feasible. A cancellation test was recommended for primary use, in cases where time allows for only a single neglect assessment (Table 2). When time permits and other tests are available, line bisection, figure copying, and the baking tray tasks were recommended for secondary use. Finally, when more extended time is available and/or when the patient's physical condition has sufficiently improved, prolonged observation with the Catherine Bergego Scale was recommended for a functional or ecological assessment, ideally by an experienced observer such as a therapist. Overall, this paper provides expert guidance for clinicians seeking to detect neglect impairment within real‐world clinical environments.

Importantly, this project aimed to recommend neglect tests that can be completed quickly for newly admitted patients in acute and subacute settings. These tests can be performed by any member of the multidisciplinary team. Ideally, the detection of neglect impairment should be based on a battery of both pen‐and‐paper and functional/ecological neglect assessments [18]. Considering results across multiple tests is extremely informative, as scores on individual tests can be expected to fluctuate due to patient alertness, time since stroke, level of distraction, spontaneous recovery, and strategic adaptation to being administered tests [5, 10, 56, 95, 96]. However, this practice is frequently perceived to be precluded by resource and time constraints associated with real‐world clinical environments. Hence, we have provided very pragmatic recommendations to use when there is time to perform only one test, and more extensive recommendations for secondary tests if further assessment is possible.

The recommended tests can be used to improve the neglect screening practice to inform patients/family members and the multidisciplinary stroke team. For example, occupational therapists play a key role in screening and supporting stroke patients with neglect, but their findings are not always taken into account by other members of the multidisciplinary team. Establishing a structured neglect screening process can help improve communication between different members of the multidisciplinary team. This practice can help to more efficiently identify each patient's individual needs and therefore provide the foundation needed to develop individualized rehabilitation programs.

One important limitation acknowledged by the panel is that there is no established, independent “ground truth” metric or gold standard for determining the presence of neglect. Given this issue, it is not possible to determine whether individual performances on any given assessment represent false positive or false negative impairment categorizations. There is also some degree of fluctuation within the results of any single test, but this does not preclude the drawing of meaningful conclusions based on screening tests. For example, impaired performance on cancellation tests, regardless of the lack of underlying ground truth, acts as a key predictor of reduced quality of life, poor functional recovery, and many other real‐world outcomes [3, 4, 5]. This relationship demonstrates the clear value of neuropsychological neglect assessments even in the absence of objective ground truth impairment categorizations. Future investigations can aim to develop potential gold standard tests for neglect. However, it is critically important for these future tests to adequately consider variation within neglect and to base all diagnostic categorizations on normative data.

Additionally, potential lack of generalizability is a key issue within the summarized neglect literature. Many tests have been administered in only a small and comparatively homogenous sample, and it is not entirely clear whether these results are adequately generalizable to the stroke population. This issue was considered when evaluating included tests, with screens supported by data from large and representative populations considered to be superior to those only tested in small groups.

### Conclusions

Overall, this study provides expert consensus recommendations on the best ways to detect neglect impairment within real‐world clinical environments. Critically, these recommendations are for rapid, preliminary neglect screening. Consideration across multiple, distinct neglect screening measures is necessary before neglect can be formally diagnosed. The panel recommends cancellation tasks for primary assessment; baking tray, figure copying, or line bisection tasks for secondary assessment, and functional neglect assessments if time allows for more in‐depth testing. These recommendations can be applied to help optimize current practice to improve neglect screening. This in turn will help provide important prognostic indicators for stroke survivors and facilitate the application of targeted neglect rehabilitation approaches.

## AUTHOR CONTRIBUTIONS

**Margaret Jane Moore:** Data curation (lead), formal analysis (lead), investigation (equal), methodology (equal), writing–original draft (equal), writing–review & editing (equal). **Elise Milosevich:** Data curation (equal), formal analysis (equal), investigation, methodology, writing–review & editing. **Roland Beisteiner:** Data curation (equal), writing–review & editing. **Audrey Bowen:** Data curation (equal), writing–review & editing. **Matthew Checketts:** Data curation (equal), writing–review & editing (equal). **Nele Demeyere:** Data curation (equal), writing–review & editing (equal). **Helena Fordell:** Data curation (equal), writing–review & editing (equal). **Oliver Godefroy:** Data curation, writing–review & editing. **Jan Laczó:** Data curation, writing–review & editing. **Timothy Rich:** Data curation, writing–review & editing. **Lindy Williams:** Data curation, writing–review & editing. **Kate Woodward‐Nutt:** Data curation, writing–review & editing. **Masud Husain:** Conceptualization (lead), data curation (equal), funding acquisition (lead), investigation, methodology, project administration (lead), resources (lead), supervision (lead), writing–review & editing.

## CONFLICT OF INTEREST

Some authors were involved in the design of neglect tests evaluated in this review. Specifically, N.D. is involved with the design, administration, and dissemination of the OCS neglect tests. M.M. and E.M. are members of N.D.'s research group. T.R. is currently employed at the Kessler Foundation, which is responsible for designing and disseminating the Kessler Foundation Neglect Assessment Procedure (KF‐NAP). H.F. is involved in design, validation, and dissemination of a digital screening test battery for Spatial Neglect, VR‐DiSTRO, for the company Brain Stimulation, partly owned by Umeå University. None of the other authors has any conflict of interest to disclose.

## DATA AVAILIBILITY STATEMENT

All data associated with this project are openly available on the Open Science Framework (https://osf.io/fzmde/).
